# Purified IgG from Patients with Obstetric but not IgG from Non-obstetric Antiphospholipid Syndrome Inhibit Trophoblast Invasion

**DOI:** 10.1111/aji.12341

**Published:** 2014-12-02

**Authors:** Katie Poulton, Vera M Ripoll, Charis Pericleous, Pier Luigi Meroni, Maria Gerosa, Yiannis Ioannou, Anisur Rahman, Ian P Giles

**Affiliations:** 1 Division of Medicine, Centre for Rheumatology, Rayne Institute, University College London (UCL)London, UK; 2 Division of Rheumatology, Department of Clinical Sciences and Community Health, Istituto G Pini, University of MilanMilan, Italy; 3 IRCCS Istituto Auxologico ItalianoMilan, Italy*; 4 Arthritis Research UK Centre for Adolescent Rheumatology, UCL, UCL Hospital and Great Ormond Street HospitalLondon, UK

**Keywords:** Antiphospholipid, obstetric, TLR4, trophoblast

## Abstract

**Problem:**

Some patients with antiphospholipid syndrome (APS) suffer pregnancy morbidity (PM) but not vascular thrombosis (VT), whilst others suffer VT only. Therefore, we compared the effects of IgG from VT+/PM− and VT−/PM+ subjects on human first-trimester trophoblast (HTR8) cells.

**Method of study:**

HTR-8 cells were incubated with APS VT+/PM−, APS VT−/PM+ or healthy control (HC) IgG. We measured trophoblast invasion by cell invasion assay; mRNA expression of TLR4 and adaptor proteins; phosphorylation of p38 MAPK, NFκB and ERK; and expression of interleukin (IL)-8 and IL-6.

**Results:**

VT−/PM+ IgG, but not VT+/PM− IgG significantly reduced HTR-8 invasion. The effects on invasion were blocked by TLR-4 inhibition. Neither VT+/PM− nor VT−/PM+ IgG altered MyD88 mRNA expression, phosphorylation of signalling molecules or cytokine expression.

**Conclusions:**

VT−/PM+ IgG exert functionally relevant effects on human trophoblast cells but VT+/PM− IgG do not.

## Introduction

Patients with the antiphospholipid syndrome (APS) have circulating antiphospholipid antibodies (aPL) which cause vascular thrombosis (VT) and/or pregnancy morbidity (PM). APS is now recognized as the most common cause of acquired hypercoagulability in the general population^1^ and the most important treatable cause of recurrent miscarriage.^2^ Despite treatment with aspirin and heparin from early pregnancy, which significantly increases the live birth rate in recurrent miscarriage patients with APS, the incidence of severe late pregnancy complications remains high^3^ and more targeted therapies are required.

It was originally thought that pregnancy complications in patients with APS were due to thrombotic events at the maternal–foetal interface. The successful use of anticoagulants in preventing foetal loss in these patients supported this notion. Histological comparison, however, of products of conception from aPL-positive and negative patients with recurrent early miscarriage has shown a specific defect in decidual endovascular trophoblast invasion in patients with APS,^4^ whereas placental infarction is not specific to patients with APS.^5^ Furthermore, aPL have been shown to have direct effects, both *in vitro* and *in vivo*, on endometrial and trophoblast cells resulting in impaired implantation and placental development (reviewed in^6^). aPL that cause PM via these non-thrombotic effects may not increase the risk of VT, and conversely, aPL that cause VT may not increase the risk of PM. This hypothesis fits with clinical observations that some patients with APS and VT never suffer PM (VT+/PM−) and some patients with APS and PM never suffer VT (VT−/PM+) despite many years of follow-up.^7^ It is therefore important to compare the biological effects of IgG derived from these patients with thrombotic and non-thrombotic APS on cultured human cells *in-vitro*.

Lopez-Pedrera et al.^8^–^10^ have demonstrated that monocytes isolated from patients with thrombotic APS have different properties compared to monocytes isolated from patients with non-thrombotic APS. In patients with thrombotic APS only, they found increased levels of circulating vascular endothelial growth factor (VEGF) and its soluble receptor Flt-1^8^ and increases in monocyte tissue factor (TF) expression, p38 mitogen-activated protein kinase (MAPK) and nuclear factor kappa B (NFκB) activation and protease activated receptor (PAR) 1 and 2 expression.^10^ Similar effects were seen in healthy volunteers' monocytes exposed to pooled IgG from patients with thrombotic APS. Supporting this observation, we have shown that IgG isolated from individual patients with thrombotic APS caused activation of p38 MAPK and NFκB signalling pathways and up-regulation of TF activity in human monocytes compared with IgG from patients with non-thrombotic APS, which lacked these effects.^11^ These effects were reduced in the presence of toll-like receptor (TLR)4 inhibitors.

In contrast, very few studies have compared the effects of thrombotic versus non-thrombotic APS-IgG in cell types relevant to PM such as trophoblast and endometrium. Mulla et al.^12^ showed that two murine monoclonal anti-β_2_ glycoprotein I (β_2_GPI) antibodies ID2 and IIC5 induced a TLR4/myeloid differentiation primary-response gene 88 (MyD88)-mediated pro-inflammatory response in the human first-trimester trophoblast line HTR-8, leading to reduced cell viability and up-regulation of interleukin (IL)-8, monocyte chemo-attractant protein (MCP)-1, growth-related oncogene (GRO)-α and IL-1β. They also demonstrated that IgG purified from patients with APS and PM stimulated trophoblast production of IL-8 and GRO-α^12^ significantly more than IgG from patients with APS but no PM (thrombosis only). In a functional assay, this group subsequently demonstrated that ID2 and IIC5 also inhibit invasion of HTR-8 cells across a membrane^13^ but did not study the effects of polyclonal IgG from patients with APS in that assay.

In this study, we report a comparison of the effects of IgG from patients with VT+/PM−, patients with VT−/PM+ and healthy control (HC) subjects on invasion of human trophoblast cells and their intracellular effects on the TLR4 pathway. TLR signalling is mediated via a family of five adaptor proteins: MyD88, MyD88-adaptor-like (MAL), toll/interleukin-1 receptor-domain-containing adaptor protein inducing interferon-β (TRIF), TRIF-related adaptor molecule (TRAM) and sterile α- and armadillo-motif-containing protein (SARM).^14^ Stimulation of TLR4 facilitates the activation of two pathways: the MyD88-dependent or MyD88-independent pathway with recruitment of TRIF and TRAM. The activation of the TLR4 MyD88-dependent pathway leads to activation of p38 MAPK and/or NFκB. Therefore, in this study, we looked at expression of TLR4, MyD88, TRIF and phosphorylation of p38 MAPK, NFκB and extracellular signal-regulated kinase (ERK). We also measured levels of the cytokines IL-8 and IL-6 as they are all important in regulating trophoblast growth and function. Furthermore, their expression has been shown to be increased when HTR-8 cells are exposed to human or murine aPL.^12^,^13^

## Materials and methods

### Patients

Serum samples from 22 individuals were obtained for this research from patients under our care at University College London Hospital, London, UK, and through Professor Silvia Pierangeli at University of Texas Medical Branch, Galveston, USA, and Professor Pier Luigi Meroni at University of Milan, Milan, Italy. All the subjects signed consent forms approved by the local ethics committees at each institution. Of 16 patients fulfilling the classification criteria for APS,^15^ nine had a history of VT alone (VT+/PM−) and seven had experienced only PM (VT−/PM+). Serum samples from six aPL-negative healthy controls (HC) were also used.

### Purification and Immunological Characterization of IgG

All polyclonal IgG was purified by protein G–Sepharose chromatography (Pierce, UK), passed through Detoxi-Gel™ Endotoxin removing columns (Pierce, UK) and subsequently determined to be endotoxin free (<0.125 Endotoxin units/mL) by the Limulus amebocyte lysate assay (Sigma, Gillingham, UK). The polyclonal APS-IgG was purified from stored serum samples that were confirmed to have aCL and anti-β_2_GPI activity. The aCL and anti-β_2_GPI activity of IgG was then measured as described previously^16^ using international calibrators in G phospholipid units (GPLU, from APL Diagnostics, Galveston, TX, USA) for the CL assay and an in-house standard of a patient with positive aPL (but no APS) with known anti-β_2_GPI binding for the anti-β_2_GPI assay [results expressed as standard units (SU)]. Pooled IgG was obtained by combining an equal concentration of IgG with similar aPL binding from five individual samples in the VT+/PM−, VT−/PM+ and HC groups shown in TableI. Two different batches, each consisting of overlapping IgG from the three comparator groups, of pooled IgG were used in these experiments.

**Table I:** Clinical and Laboratory Features of Patients and Controls

	VT+/PM− (*n* = 9)	VT−/PM+ (*n* = 7)	HC (*n* = 6)
Age (mean ± SEM)	53.3 ± 5.9	43.4 ± 1.9	33.5 ± 3.7
Sex	6 F/3 M	7 F	6 F
PAPS	6 (66.6%)	6 (85.7%)	0
SLE	3 (33.3%)	1 (14.3%)	0
No. pregnancies	4	24	3
Live births	4	16	3
Total APS-related PM	0	7 (6 ST-PL, 1 TT-PL)	0
Arterial thrombosis	5 (3 CVA, 2 TIA)	0	0
Venous thrombosis	5 (4 DVT, 4 PE)	0	0
Plasma LA positive	8	6	NT
Serum aCL (mean GPLU ± SEM)	144.3 ± 23.4	120.9 ± 14.6	4.3 ± 0.8
Serum anti-β_2_GPI (mean SU ± SEM)	83.3 ± 17.3	84.4 ± 28.9	0.2 ± 0.1
IgG aCL (mean GPLU ± SEM)	86.5 ± 19.7	65 ± 10.6	0 ± 0
IgG anti-β_2_GPI (mean SU ± SEM)	61.3 ± 26.3	71.8 ± 38	0 ± 0

aCL, anti-cardiolipin antibodies; anti-β_2_GPI, anti-β_2_-glycoprotein I antibodies; CVA, cerebrovascular accident; DVT, deep vein thrombosis; F, female; GPLU, IgG phospholipid units; LA, lupus anticoagulant; M, male; NT, not tested; PAPS, primary antiphospholipid syndrome; PM, pregnancy morbidity; PE, pulmonary embolus; CVA, cerebrovascular accident; SEM, standard error of the mean; SLE, systemic lupus erythematosus; ST-PL, second-trimester pregnancy loss; SU, standard units; TIA, transient ischaemic attack; TT-PL, third-trimester pregnancy loss.

Lupus anticoagulant activity was checked on plasma samples by dilute Russell viper venom time and activated partial thromboplastin time. Purified IgG was tested at the final experimental concentration of 100 μg/mL.

### First-trimester Trophoblast Cell Line

The human first-trimester extravillous trophoblast cell line HTR-8, immortalised by SV40,^17^ was kindly provided by Dr Charles Graham from Queen's University, Kingston, Ontario, Canada. HTR-8 cells were cultured in RPMI 1640 (Gibco), supplemented with 10% foetal bovine serum (PAA Laboratories, GE Healthcare Life Sciences, Bucks, UK) and 100 units/mL penicillin 100 μg/mL streptomycin (Gibco, Paisley, UK) and maintained at 37°C/5% CO_2_. Cells were treated with 100 μg/mL of a pool of either VT+/PM− IgG, VT−/PM+ IgG or HC-IgG. Each IgG pool was derived from five individual patients or controls. In some instances, HTR-8 cells were pre-treated for 1 hr with 1 μm of the TLR4 inhibitor CLI-095 (InvivoGen, Toulouse, France), which blocks the signalling mediated by the intracellular domain of TLR4 or 1 μg/mL of Ultra Pure *Rhodobacter sphaeroides* LPS (InvivoGen), a TLR4 antagonist that does not induce TLR4 signalling.

### Trophoblast Cell Invasion Assay

The QCM 24-well collagen-based cell invasion assay (Chemicon International, Temecula, CA, USA) was used to compare the ability of HTR-8 cells incubated with APS-IgG or HC-IgG to invade through a collagen layer. In short, invasion chamber inserts containing a collagen layer above a polycarbonate membrane were placed into wells of a 24-well tissue culture (TC) plate. 1.25 × 10^5^ HTR-8 cells in a total volume of 300 μL were added to each invasion assay insert and 500 μL of RPMI were added to the well of the TC plate outside the insert. Pooled APS-IgG or HC-IgG (100 μg/mL) was added to separate invasion chamber inserts. Following 48 hr incubation (a time point selected based on previous similar studies^13^), each invasion chamber insert was removed from its TC well and the non-invading cells/media from the top of the insert were removed. The cells that had invaded through the collagen layer to attach to the polycarbonate membrane were collected and stained with a dye. The amount of dye retained is a measure of the number of cells that invaded through the collagen layer and was assayed by transferring samples to 96-well plate and reading optical density on a TECAN GENios Microplate Reader at 560 nm. The percentage of cells that invaded when cells were incubated with APS-IgG were calculated relative to an invasion control where HC-IgG was added which was considered to have 100% invasion.

### qRT-PCR

Following 6 hr incubation with 100 μg/mL pooled APS-IgG or HC-IgG, total RNA was isolated from HTR-8 cells using phenol–chloroform extraction. The expression of *TLR4, TRIF, MyD88, IL-8* and *IL-6* mRNA was measured by qRT-PCR using TaqMan probes (Applied Biosystems, Paisley, UK). Samples were run on a DNA Engine Opticon continuous fluorescence detector (MJ Research) under the following conditions: initial denaturation: 95°C for 10 min, followed by 41 cycles of: 95°C for 15 s, 60°C for 1 min. Gene expression was determined relative to the housekeeping glyceraldehyde 3-phosphate dehydrogenase (*GAPDH*) gene mRNA using the comparative cycle threshold (*C*_t_) method. Results are expressed as fold change relative to untreated cells.

### Immunoblot

Following 15 min incubation of HTR-8 cells with pooled APS-IgG or HC-IgG (100 μg/mL), cell extracts were prepared by addition of 100 μL lysis buffer [50 mm Tris–HCl pH 7.4, 150 mm NaCl, 5 mm EDTA, 1 mm EGTA, 1% NP-40, 0.1% SDS, 0.5% NA-Deoxycholate, 10 mm NaF, 1 mm Na_3_VO_4_, and complete mini protease inhibitor cocktail tablet; Roche, Welwyn Garden City, UK]. Cell lysate (20 μg) was resolved on a 10% sodium dodecyl sulphate polyacrylamide gel electrophoresis under reducing conditions, transferred to nitrocellulose membranes, blocked with 5% BSA and incubated overnight at 4°C with primary antibody – rabbit anti-human phosphorylated p38 MAPK (Thr^180^/Tyr^182^), total p38 MAPK, phosphorylated NFκB p65 (Ser^536^), total NFκB p65, phosphorylated p44/42 MAPK (Thr^202^/Tyr^204^) or total ERK1 (Cell Signalling, Danvers, MA, USA) – followed by 1-hr incubation in 1:2000 dilution of horseradish peroxidase-conjugated goat anti-rabbit IgG (Dako, Ely, UK). Phosphorylated and total protein for the same signalling protein were analysed on the same membrane. After detection of the phosphorylated protein, the antiphosphorylated protein antibody was removed by 0.2 M sodium hydroxide and blocked with 5% BSA. After removal of the antibody specific for phosphorylated protein, membranes were incubated overnight with antibody to the total protein and the process was repeated. Protein bands were visualized by chemiluminescence (GE Healthcare, Amersham, UK) and their intensity quantified by densitometric analysis (QuantityOne software; Biorad, Hemel Hempstead, UK), and results were expressed as a ratio of relative expression.

### Cytokine ELISA

HTR-8 cells were incubated with pooled APS-IgG or HC-IgG (100 μg/mL) for a range of time periods from 2 to 72 hr. The cell culture supernatant was collected by centrifugation at 400 ***g*** for 10 min and stored at −80°C. IL-8 and IL-6 were measured using commercially available ELISA kits (IL-8 BD Biosciences, Oxford, UK and IL-6 R&D systems, Abingdon, Ox, UK). Assays were performed following the manufactures instructions. Detection and analysis were performed using the TECAN GENios Microplate Reader (Reading, UK).

### Statistics

For each outcome, the experiments were repeated at least three times independently and data are expressed as mean ± the standard error of the mean (SEM) of these triplicates. Statistical analysis was undertaken using one-way analysis of variance (anova) – Kruskal–Wallis test – with Duns multiple post hoc comparison and assessed for overall statistical significance at the 5% level (*P* < 0.05). Data analysis was performed using the GraphPad Prism software program (GraphPad Software, San Diego, CA, USA).

## Results

### Clinical and Laboratory Characteristics of Subjects

The clinical and laboratory characteristics of the 16 patients with APS (9 VT+/PM− and 7 VT−/PM+) and 6 HC subjects are outlined in TableI. Nineteen (86.4%) of the 22 subjects in this study were women. Of the 16 patients with APS (both VT+/PM− and VT−/PM+) four patients have SLE/APS and 12 have primary APS. Of the patients with VT+/PM−, four had venous thrombosis, four arterial thrombosis and one patient experienced both arterial and venous thrombosis. Of the patients with VT−/PM+, six had experienced a second-trimester foetal loss and 1 a third-trimester foetal loss, all fulfilling APS PM classification criteria.^15^ Both serum and purified IgG from patients with APS had significantly higher aCL and anti-β_2_GPI activity compared to HC. The comparable levels of individual purified IgG aCL and IgG anti-β_2_GPI (tested at the final experimental concentration of 100 μg/mL) in the VT+/PM− (86.5 GPLU and 61.3 SU respectively) and VT−/PM+ (65 GPLU and 71.8 SU respectively) groups indicate that any differences in the functional effects of IgG from these two groups are unlikely to be due to differences in levels of aPL. The levels of aCL and anti-β_2_GPI activity in each pool are shown in the relevant figure legend.

### IgG Purified from Patients with Obstetric APS Inhibit Trophoblast Invasion in a TLR4-Dependent Manner

**Figure 1:**
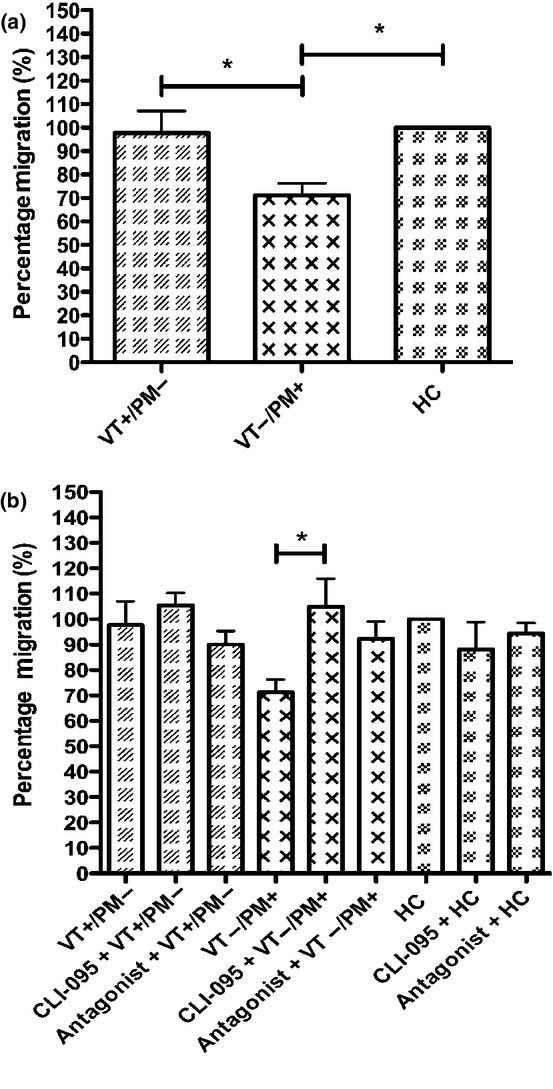
IgG purified from patients with VT−/PM+ APS inhibit HTR-8 cell invasion, which is abrogated when cells are treated with a TLR4 inhibitor. The ability of HTR-8 cells to invade after treatment with 100 μg/mL pooled IgG from VT+/PM− with 83.7 GPLU and 66.0SU binding activity, VT−/PM+ with 47.2GPLU and 63.8SU binding activity and HCs with 0GPLU and SU binding (a) and following pre-treatment with the TLR4 inhibitor CLI-095 or TLR4 antagonist, Ultra Pure *Rhodobacter sphaeroides* LPS (b) was measured using a transwell invasion assay after 48 hr. HC cell invasion was set at 100%, and the relative invasion of HTR-8 cells exposed to APS-IgG was analysed from this. Graph shows mean ± SEM of quantitative analysis from six (a) and three (b) independent experiments. Statistical analysis was performed as follows: (a) one-way anova (*P* = 0.01) with Dunn's multiple comparisons test (*P < 0.05); (b) one-way anova (*P* = 0.03) with Dunn's multiple comparisons test (*P < 0.05).

A significant (*P* < 0.05) reduction was observed in the invasion of HTR-8 cells exposed to VT−/PM+ IgG compared to HTR-8 exposed to VT+/PM− IgG and HC-IgG (Fig.1a). This inhibition was shown to be TLR4 dependent (Fig.1b) as it was reversed by pre-treatment of HTR-8 cells with the TLR4 inhibitor CLI-095 or TLR4 antagonist Ultra Pure *Rhodobacter sphaeroides* LPS restored the invasion of cells treated with VT−/PM+ IgG although only the effect of CLI-095 reached statistical significance (*P* < 0.05).

### IgG Purified from Patients with Obstetric APS Induce HTR-8 Cell mRNA Expression of TLR4 and TRIF but not MyD88 TLR Adaptor Proteins

**Figure 2:**
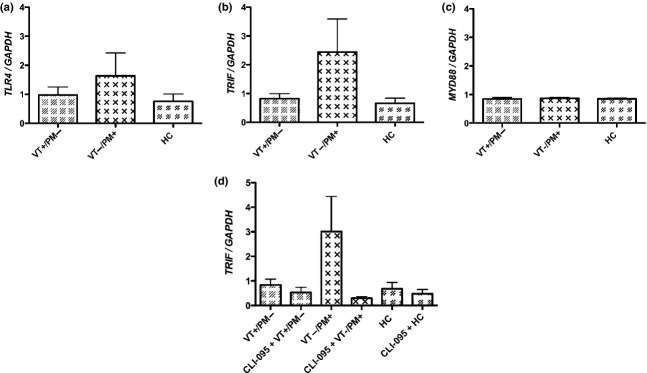
HTR-8 cells treated with VT−/PM+ IgG but not HTR-8 cells treated with VT+/PM− IgG increase TLR4 and TRIF transcript levels. HTR-8 cells were treated with 100 μg/mL pooled IgG from VT+/PM− with 78.2GPLU and 44.4SU binding activity, VT−/PM+ with 83.4GPLU and 71.5SU binding activity and HCs with 0GPLU and SU binding. *TLR4* (a), *TRIF* (b) and *MyD88* (c) mRNA expression at 6 hr was measured by qRT-PCR. *TRIF* mRNA expression in HTR-8 cells pre-treatment with the TLR4 inhibitor CLI-095 before treatment with 100 μg/mL pooled IgG from VT+/PM−, VT−/PM+ and HCs was also measured (d). The mean ± SEM of quantitative analysis from four independent experiments is shown. Results are expressed as fold change of either *TLR4, TRIF* or *MyD88*/*GAPDH*, relative to untreated cells. No statistically significant differences were found by one-way anova.

HTR-8 cells treated with VT−/PM+ IgG increased *TLR4* mRNA expression by 2.2-fold (Fig.2a) and *TRIF* mRNA expression by 3.7-fold (Fig.2b) compared to HTR-8 cells treated with HC-IgG, although these values were not statistically significant. VT−/PM+ IgG had no effect on *MyD88* mRNA expression (Fig.2c). In contrast, VT+/PM− IgG had no effect on expression of any of these mRNAs. Fig.2d shows that pre-treatment with the TLR4 inhibitor CLI-095 abrogated the increased *TRIF* mRNA expression seen in HTR-8 cells treated with VT−/PM+ IgG, although this difference failed to reach statistical significance.

### IgG Purified from Patients with APS do not Promote the Phosphorylation of p38 MAPK, NFκB p65 or ERK or the Production of the Cytokines IL-8 or IL-6 in HTR-8 Cells

**Figure 3:**
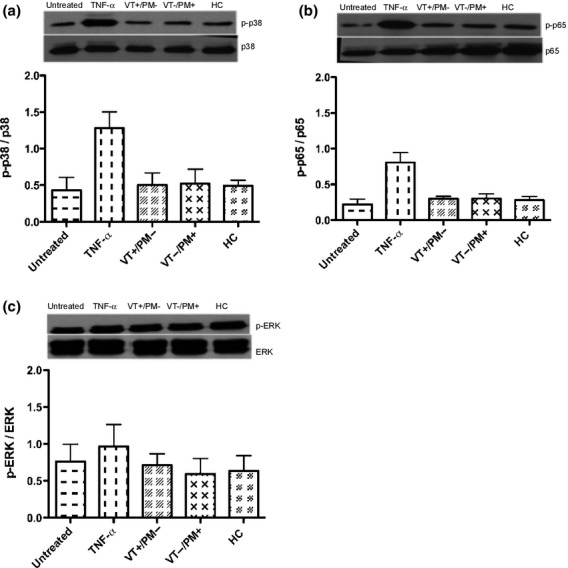
APS-IgG do not increase the phosphorylation of p38 MAPK, NFκB p65 or ERK. HTR-8 cells were treated with 100 μg/mL pooled IgG from VT+/PM− with 78.2GPLU and 44.4SU binding activity, VT−/PM+ with 83.4GPLU and 71.5SU binding activity and HCs with 0GPLU and SU binding as well as the positive controls TNF-α (10 ng/mL). Graphs show relative expression at 15 min of p38 MAPK (a), NFκB p65 (b) and ERK (c). The mean ± SEM of quantitative analysis from three independent experiments is shown. No statistically significant differences were found by one-way anova.

We then measured whether the APS-IgG-mediated stimulation of TLR4 led to preferential phosphorylation of MyD88-dependent (p38 MAPK, NFκB p65 or ERK) pathways in HTR-8 cells. Fig.3a–c shows that neither VT+/PM− IgG nor VT−/PM+ IgG increase the phosphorylation of p38 MAPK, NFκB p65 or ERK in HTR-8 cells compared to that seen in untreated cells.

**Figure 4:**
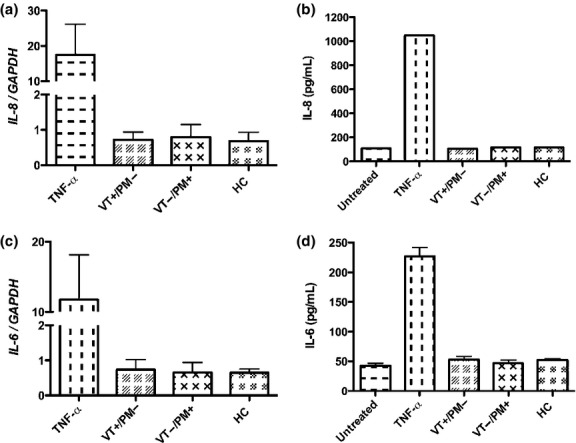
APS-IgG do not increase the transcript or protein secretion of the cytokines IL-8 and IL-6 in HTR-8 cells. HTR-8 cells were treated with 100 μg/mL pooled IgG from VT+/PM− with 83.7 GPLU and 66.0SU binding activity, VT−/PM+ with 47.2GPLU and 63.8SU binding activity and HCs with 0GPLU and SU binding as well as the positive controls TNF-α (10 ng/mL). Graphs (a–d) show qRT-PCR analysis of *IL-8* (a), and *IL-6* (c) mRNA expression at 6 hr and ELISA results of IL-8 (b) and IL-6 (d) protein expression at 72 hr. The mean ± SEM of quantitative analysis from four independent experiments is shown. No statistically significant differences were found by one-way anova.

We also investigated HTR-8 cell expression of IL-8 and IL-6, following incubation with APS-IgG and HC-IgG utilising both qRT-PCR and ELISA. There was no difference in the mRNA or protein expression of any of these cytokines in HTR-8 cells treated with VT+/PM− IgG, VT−/PM+ IgG or HC-IgG at any time point from 2 to 72 hr. Fig.4a–d shows the results for mRNA at 6 hr and protein expression at 72 hr (the time points where mRNA and protein expression respectively were maximal in cells exposed to TNF-α, the positive control).

## Discussion

This study has shown that only IgG from patients with obstetric (non-thrombotic) APS inhibit trophoblast invasion *in-vitro* in a TLR4-dependent manner, compared with thrombotic APS-IgG which lacked this effect. Furthermore, only obstetric APS-IgG increased the transcript expression of *TLR4* and *TRIF*, although this difference failed to reach statistical significance. However, neither VT+/PM− nor VT−/PM+ IgG had any effect on *MyD88* mRNA expression, phosphorylation of the p38 MAPK, NFκB or ERK signalling pathways or expression of IL-8 and IL-6 in this experiment.

These results can be compared with our previous work on human monocytes.^11^ In those cells, we found the reverse, that is, that only thrombotic (VT+/PM−) IgG had a functionally important effect (of increased TF activity), whereas VT−/PM+ IgG had no effect. Although that effect on monocyte TF activity was also reduced by TLR4 inhibition, it seems likely to have been mediated via different signalling pathways to those involved in the HTR-8 cells because we observed increased phosphorylation of the p38 MAPK and NFκB signalling pathways in monocytes treated with VT+/PM− IgG. Currently, the mechanism of the TLR4-dependent effect on HTR-8 cells demonstrated in our experiment is unclear.

The inhibitory effects of APS-IgG upon the invasion of trophoblast cells *in-vitro* are well-documented.^13^,^18^–^20^ The mechanisms underlying these inhibitory effects are likely to be complex and are not fully understood. In an important series of papers, Mulla and colleagues used the murine monoclonal anti-β_2_GPI antibodies ID2 and IIC5 to demonstrate both TLR4-dependent and TLR4-independent effects on human trophoblast cells. Thus, in HTR-8 cells, these antibodies cause TLR4-dependent up-regulation of the inflammatory cytokines IL-8, MCP-1, GRO-α and IL-1β^12^ but TLR4-independent reduction of IL-6 secretion and signal transducer and activator of transcription (STAT)-3 activation.^13^ They also showed that these monoclonal antibodies inhibit HTR-8 invasion but did not investigate whether that effect was TLR4-dependent (although it was IL-6-dependent). ID2 and IIC5 also cause TLR4-independent but MyD88-dependent secretion of angiogenic factors, which is not reversed by heparin.^21^ Most recently, this group showed that these two murine monoclonal antibodies stimulate production of uric acid in a different trophoblast line (Sw.71) in a TLR4-dependent manner, which activates the inflammasome and promotes IL-1β processing and secretion.^22^

Comparing our results using polyclonal human anti-β_2_GPI antibodies with those of Mulla et al. using xenogenic anti-β_2_GPI monoclonals, we have observed many complementary findings. We have demonstrated a TLR4-dependent mechanism of inhibition of HTR-8 invasion by human APS-IgG, which may operate in addition to the TLR4-independent, IL-6-dependent inhibition described by Mulla's group. As we did not use dominant-negative MyD88 transfection vectors, we cannot be sure whether this inhibition acts via MyD88. Given our findings, however, we suspect that it is more likely to operate via the TRIF/TRAM pathway, although we were unable to optimise experiments to confirm increased TRIF expression at the protein level. Previous work examining the involvement of TLR adaptor proteins in APS-mediated cell signalling in other non-obstetric cell types focussed upon the MyD88 pathway. Human anti-β_2_GPI antibodies have been shown to activate endothelial cells in a MyD88-dependent manner through involvement of TLRs.^23^ Studies of the human monocytic THP-1 cell line have shown that incubation with rabbit anti-β_2_GPI/β_2_GPI complexes increased *TF* mRNA expression, TF activity, and expression of TLR4, MyD88, and myeloid differentiation protein-2, a critical cofactor that interacts with TLR4.^24^ Recently, the same group showed that treatment of healthy *ex vivo* monocytes or THP-1 cells with monoclonal anti-β_2_GPI/β_2_GPI complexes increased MyD88 and TRIF mRNA and protein expression and this effect was blocked by addition of TAK-242, a blocker of signalling transduction mediated by the intracellular domain of TLR4.^25^ To our knowledge, we are the first to identify the involvement of TRIF in APS TLR4-mediated trophoblast signalling.

It is difficult, however, to make a direct comparison between experiments using xenogenic monoclonal and spontaneous autoimmune anti-β_2_GPI antibodies, because obvious differences exist such as epitope specificity and avidity. The strongest evidence for pathogenicity relates to antibodies against β_2_GPI, which has five domains (DI-DV), particularly those antibodies directed against DI. We^26^ and other groups^27^–^31^ have shown that circulating levels of IgG aDI are elevated in patients with APS in comparison with healthy and disease controls. The xenogenic anti-β_2_GPI monoclonals described above have been shown to recognise epitopes in DV^32^ whilst 7 of 16 APS-IgG used in our study bind DI. The clinical association and titre of anti-DI binding of serum samples in this study are shown in Table S1. Four of the VT+/PM− and three of the VT−/PM+ samples are aDI positive. None of the samples were tested for binding to other domains of β_2_GPI, as their significance is yet to be established in APS, and they are not currently being developed as non-criteria assays for clinical use in APS.^33^

A potential disadvantage, however, of using polyclonal compared with monoclonal IgG is that the observed biological effect may attributed to another autoantibody population such as may be found in patients with SLE. We do not believe, however, that the inclusion of SLE/APS in our pooled samples has adversely affected our results for several reasons. First, the clinical and serological features of APS and SLE associated APS are known to be similar.^1^,^34^ Second, we have previously shown that whereas IgG from patients with APS (with or without SLE) stimulated phosphorylation of NFkB or p38MAPK and increased TF activity in human monocytes compared with healthy control IgG, purified IgG from aPL-positive patients with SLE but no APS (APL+/APS−) did not stimulate any of these effects in monocytes. This lack of effect was seen despite the fact that nine of 12 patients from this aPL+/APS− group had a range of other serum autoantibodies, such as anti-dsDNA and anti-Ro.^11^ These differences between monoclonal and polyclonal IgG may partly explain why, in contrast to previous papers, we did not demonstrate effects of APS-IgG on secretion of the cytokines IL-8 or IL-6 in HTR-8 cells. Notably, the absolute levels of IL-6 (both mRNA and protein) in cells exposed to APS-IgG in our experiment were similar to those described by Mulla et al.^12^,^13^ The difference in our experiment was the inclusion of cells exposed to HC-IgG and as these cells also secreted IL-8 or IL-6 at similar levels we were unable to confirm a specific effect of the APS-IgG samples on these outcomes.

Trophoblast invasion and successful implantation *in vivo* depend on a complex series of molecular and cellular events that are induced in the pregnant uterus by various paracrine and autocrine regulators in addition to cytokine release.^35^ Therefore, future experiments will require measurement of these factors in response to IgG purified from patients with only thrombotic or obstetric APS in both *in vitro* and *in vivo* models. In particular, LPS-mediated stimulation of macrophages undergoing endoplasmic reticulum stress has recently been shown to produce mature IL-1β via TLR4, caspase-8 and TRIF-dependent signalling pathways.^36^ Therefore, it will be important to address the potential involvement of caspase 8 and other APS-IgG-mediated signalling in HTR8 and primary trophoblast cells.

We used two different batches of pooled IgG created from five individuals for the APS-IgG and HC-IgG preparations. Our use of pooled samples enabled us to test the different comparison groups against a large number of biological outcomes on trophoblast cells which would not have been possible if we had tested multiple samples from each of the studied groups. In creating these different batches of pooled IgG for our experiments, it proved impossible to produce a second batch of all-female IgG with similar aPL binding between aPL subgroups. Therefore, it was necessary to include three male samples. Interestingly, monoclonal IgG aCL from male patients has been shown to induce foetal loss in naive mice as well as the IgG from female patients with obstetric APS.^37^ Therefore, we do not believe that inclusion of these male samples has adversely affected our findings, although future experiments will include an all-female cohort.

Similarly, we were unable to match our comparator groups for parity. Interestingly, many previous, similar studies of the effects of aPL upon trophoblast cells have not matched parity between test and control groups. For instance, Mulla et al.^12^ and Carroll et al.^21^ examined the same cohort of (*n* = 6) VT+/PM− patients with three live births from six pregnancies and (*n* = 6) VT−/PM+ patients with six live births from 22 pregnancies; Bose et al.^19^ examined (*n* = 3) LA-positive patients with recurrent miscarriage with a mean number of live births of 0.3 (range 0–1) from a mean number of first-trimester miscarriages was 4.2 (range 3–8) and (*n* = 3) control human sera with an undisclosed number of pregnancies; Jovanovic et al.^20^ examined (*n* = 13) patients with APS and (*n* = 10) control human sera with an undisclosed number of pregnancies; and Mulla et al.^22^ examined (*n* = 55) aPL+ patients with 10 of 55 adverse pregnancy outcomes compared with (*n* = 113) healthy controls of whom four had an adverse pregnancy outcome. In contrast, di Simone et al.^18^ were able to match parity in their examination of (*n* = 2) APS samples with no live births from five pregnancies and (*n* = 2) aPL+/APS− samples with four live births from four pregnancies.

In our cohort, we have found that women in the VT+/PM− group are less likely to want to become pregnant because they are on warfarin and also have an appreciable risk of DVT in pregnancy, so it is intrinsically difficult to match parity in these groups. This finding of reduced parity in patients with SLE is well known and one study of (*n* = 119) women with SLE found that they viewed their disease as a barrier to childbearing^38^ and another multicentre study found that 42% of (*n* = 339) women with SLE diagnosed before 50 years of age had never been pregnant.^39^ Overall, we do not believe that the lack of parity between groups in our and other similar studies will have had a significant influence upon our findings.

None of the samples available to us at the time of testing were derived from patients with recurrent first-trimester miscarriage fulfilling APS criteria; thus, we were unable to compare effects of IgG from patients with early and late APS-related pregnancy loss. In fact, two patients with VT−/PM+ had experienced a single first-trimester miscarriage, although we cannot be certain that these events were APS-related so have not included this clinical information in TableI. Interestingly, the recent obstetric APS taskforce identified that although a majority of studies report a positive association between aPL and recurrent early miscarriage these studies are highly heterogenous regarding clinical events and laboratory criteria, so very few actually meet APS classification criteria.^40^ This taskforce also noted heterogeneity in studies of late pregnancy manifestations but found a stronger association between late pregnancy morbidity (foetal death, pre-eclampsia, IUGR) and aPL from more recent multicentre prospective studies and an association with double/triple aPL positivity and higher titres of aPL. Therefore, selection of samples with late pregnancy APS manifestations may actually be advantageous as this aPL profile is most likely to have comparable aPL titres with thrombotic APS samples which usually display the highest titres as was the case in our cohort. In future work, however, it will be important and to select VT−/PM+ patients with recurrent first-trimester miscarriage, to confirm that they have a similar effect upon trophoblast cells to the samples with late pregnancy morbidity that we studied.

In classifying our patients with VT+/PM− or VT−/PM+, we cannot completely exclude the possibility that a patient who has previously suffered only VT may subsequently develop PM or vice versa. Patients in the VT−/PM+ group, however, with a mean age of 43.1 years have been followed up for many years with no VT event (>10 years for all UCLH patients). Of the six women in the VT+/PM− group, four had never been pregnant (for reasons of personal choice). The other two had two normal pregnancies each. Therefore, although some of the patients with VT+/PM− could theoretically be misclassified (as they could have had PM if they had ever been pregnant), such misclassification of patients would have reduced our chance of being able to distinguish a difference between groups rather than leading to false-positive differences. In future experiments, it would be important to repeat this work using samples from individual patients and to study the effects of these different groups of IgG upon maternal/decidual cells.

In summary, we have identified that IgG isolated from patients with APS-related PM preferentially inhibit the invasion of a human trophoblast cell line compared with APS-IgG from patients with VT alone. This effect is dependent on TLR4. Further experiments are now required to characterize the mechanistic and prognostic implications of these findings.

## Supporting Information

Table S1 Summary of IgG anti-DI activity in the sera of 16 patients with APS.
