# Supplementary material for: Purified IgG from Patients with Obstetric but not IgG from Non-obstetric Antiphospholipid Syndrome Inhibit Trophoblast Invasion
Source: Am J Reprod Immunol. 2014 Dec 2;73(5):390–401. doi: 10.1111/aji.12341 (PMC4409084; doi:10.1111/aji.12341)
Supplement: Table S1 — Summary of IgG anti-DI activity in the sera of 16 patients with APS. [file aji0073-0390-sd1.docx]

| Patient no. | Sex (F/M) | Clinical history | Serum GDIU |
| --- | --- | --- | --- |
| 1 | F | VT+/PM- | 4.9 |
| 2 | F | VT+/PM- | 19.9 |
| 3 | F | VT+/PM- | 4.7 |
| 4 | F | VT+/PM- | 7.0 |
| 5 | F | VT+/PM- | 12.5 |
| 6 | F | VT+/PM- | 8.4 |
| 7 | M | VT+/PM- | 85.8 |
| 8 | M | VT+/PM- | 100 |
| 9 | M | VT+/PM- | 4.3 |
| 10 | F | VT-/PM+ | 7.2 |
| 11 | F | VT-/PM+ | 21.0 |
| 12 | F | VT-/PM+ | 9.3 |
| 13 | F | VT-/PM+ | 5.3 |
| 14 | F | VT-/PM+ | 5.5 |
| 15 | F | VT-/PM+ | 69.3 |
| 16 | F | VT-/PM+ | 77.1 |

**Supplementary Table 1:** Summary of IgG anti-DI activity in the sera of 16 patients with APS. Activity is defined as IgG anti-DI units (GDIU). Cut off for positivity was determined to be 10GDIU based on the mean activity +3SD of 200 healthy control sera.
